# Growth-Dependent Paenibacillin Biosynthesis and Its Association with an Agr-like Quorum Sensing System in *Paenibacillus polymyxa*

**DOI:** 10.3390/antibiotics15100976

**Published:** 2026-10-03

**Authors:** Sochina Ranjit, Ahmed G. Abdelhamid, Ahmed E. Yousef

**Affiliations:** 1 Department of Food Science and Technology, The Ohio State University, 2015 Fyffe Road, Columbus, OH 43210, USA; ranjit.5@buckeyemail.osu.edu (S.R.); yousef.1@osu.edu (A.E.Y.); 2 Department of Food Science and Human Nutrition, Michigan State University, East Lansing, MI 48824, USA; 3 Department of Microbiology, The Ohio State University, 484 West 12th Avenue, Columbus, OH 43210, USA

**Keywords:** *Paenibacillus polymyxa*, paenibacillin, quorum sensing, genetic regulation

## Abstract

Background: Antimicrobial resistance in foodborne pathogens and demand for clean-label foods highlight the need for natural antimicrobial preservatives. Paenibacillin, an antimicrobial peptide produced by *Paenibacillus polymyxa* OSY-DF, has potential as a natural preservative, but low productivity limits its application. Paenibacillin is encoded by an 11-gene biosynthetic gene cluster (BGC) containing seven biosynthetic genes and a putative accessory gene regulator (Agr)-like quorum sensing (QS) locus (*agr*ABCD). Understanding the association between QS systems and paenibacillin biosynthesis may provide insights into factors influencing its production. Methods: The Agr-like QS system was assessed through a time-course transcriptional analysis of the paenibacillin BGC and antimicrobial bioassays. AI-2 activity was detected using *Vibrio harveyi*, and its potential regulatory role was assessed by inhibiting AI-2 signaling with D-ribose. Results: Transcription of *agr*ABCD increased after 4 h, reaching 18.59- to 202.61-fold upregulation at 24 h relative to 0 h. In contrast, limited transcription of the seven biosynthetic genes was detected during the initial 8 h, followed by 19.64- to 136.12-fold upregulation at 24 h. Anti-*Listeria* activity was first detected at 12 h and increased through 24 h. These transcriptional and phenotypic patterns suggest a potential association between the Agr-like QS system and paenibacillin BGC expression. AI-2 was detected in OSY-DF culture supernatants; however, reducing AI-2 signals did not reduce paenibacillin activity. Conclusions: Paenibacillin production is growth phase-dependent and temporally associated with Agr-like gene transcription, whereas AI-2 does not appear to regulate its biosynthesis. Functional validation through targeted *agr* disruption is required to define the direct regulatory role of the Agr-like QS system.

## 1. Introduction

The rise in resistance to currently used antimicrobial preservatives among foodborne pathogens may compromise food safety and pose a significant threat to public health. Foodborne pathogens of major concern, such as *Salmonella enterica*, *Escherichia coli*, and *Listeria monocytogenes*, may adapt to synthetic preservatives and chemical decontaminants [1,2,3]. In some cases, this adaptation has resulted in cross-protection against clinically important antibiotics. Exposure to sodium nitrite and acetic acid, for example, has been linked to tetracycline resistance in *S. enterica*, whereas lactic acidic stress has decreased the susceptibility of *L. monocytogenes* to the antibiotics penicillin and ciprofloxacin [1,3,4,5]. Alternative antimicrobial interventions are therefore of interest for reducing the burden of antimicrobial resistance in food microbiota. Such alternative approaches include using bacteriophages, protective cultures, plant-derived antimicrobials, and antimicrobial peptides (AMPs). The emerging interest in AMPs, particularly bacteriocins, is rational as these may serve as an effective control measure against microorganisms that are currently resistant to conventional antimicrobials [6].

Paenibacillin, a novel and potent antimicrobial peptide, is a class I bacteriocin that is naturally produced by *Paenibacillus polymyxa* OSY-DF, a bacterium that was isolated originally from kimchi [7]. This AMP is effective against Gram-positive bacteria, including *Listeria* spp., *Pediococcus cerevisiae*, *Staphylococcus aureus, Bacillus* spp., and *Clostridium sporogenes* [7]. Previous work investigating the mechanism of action of paenibacillin showed that it depolarized the bacterial cell membrane and induced potassium ion release from *S. aureus* and *L. innocua*, suggesting that membrane disruption contributes to its antibacterial activity [8]. Similar membrane-targeting mechanisms have been reported for other lantibiotics; for example, nisin interacts with lipid II as a docking molecule for membrane pore formation [9]. Together, its broad activity against Gram-positive bacteria and membrane-targeting effects support further evaluation of paenibacillin as a potential food biopreservative. On a different front, consumers are increasingly evading foods with synthetic chemical preservatives and favoring minimally processed foods with natural ingredients, including antimicrobial peptides [6,10]. Being a naturally produced potent bacteriocin, paenibacillin may be favored as an alternative to currently used synthetic food preservatives.

Native production of some AMPs, including paenibacillin by *P. polymyxa* OSY-DF, may be insufficient for large-scale production. This presents a hurdle to implementing AMPs industrially. Previous attempts to increase the production of paenibacillin were aimed at concentrating paenibacillin using a foam separation technique in a bioreactor setup [11] and creating a spontaneous mutant (*P. polymyxa* OSY-EC) based on ribosomal engineering [12]. These methods increased the production of paenibacillin by 5- to 50-fold. Despite these advances, little is known about the genetic regulations governing paenibacillin biosynthesis. Additionally, the mutant, *P. polymyxa* OSY-EC, has a slow growth rate compared with the wild-type *P. polymyxa* OSY-DF, and the long-term stability of such a spontaneous mutant would require further investigation [13]. Alternatively, defining the regulatory mechanism in the paenibacillin biosynthesis pathway might be a useful strategy that leads to increasing the production of this peptide and similar AMPs. In addition, understanding these mechanisms may provide broader insights into the regulation of lantibiotic biosynthesis and microbial secondary metabolite production.

The biosynthesis of many secondary metabolites and antimicrobial peptides in Gram-positive bacteria could be regulated by quorum sensing (QS) systems [14,15]. Such systems in *Paenibacillus* species, particularly *P. polymyxa*, remain largely uncharacterized. Some bacterial species have multiple QS systems that can be used to communicate between themselves and others [16,17]. The autoinducer-2 (AI-2) molecules, which are furanose-based structures, has been implicated in cell–cell signaling in different bacterial species [18]. The AI-2 family has been known to regulate secondary metabolites linked to biofilm production, motility, pathogenicity, and virulence [19]. Gram-positive bacteria may also have oligopeptide-based QS systems. Such systems include the accessory gene regulator (Agr) and its homologs in *Staphylococcus*, *Listeria*, and *Paenibacillus* species, which regulate biofilm formation, virulence, and other cellular mechanisms [17,20,21]. Similarly, some *Bacillus* and *Streptococcus* species carry the competence (Com) system, which regulates genetic competence and DNA uptake [22]. In some Gram-positive species, a family of protein receptors (RNPP) are involved in self-signaling pathways that control group behaviors such as sporulation, virulence, biofilm formation, conjugation, and production of extracellular enzymes. [14,20]. Within the peptide-based QS systems in Gram-positive bacteria, the Agr system in *Staphylococcus* spp. is one of the well-studied autoinducing regulatory systems [20].

**Figure 1:**
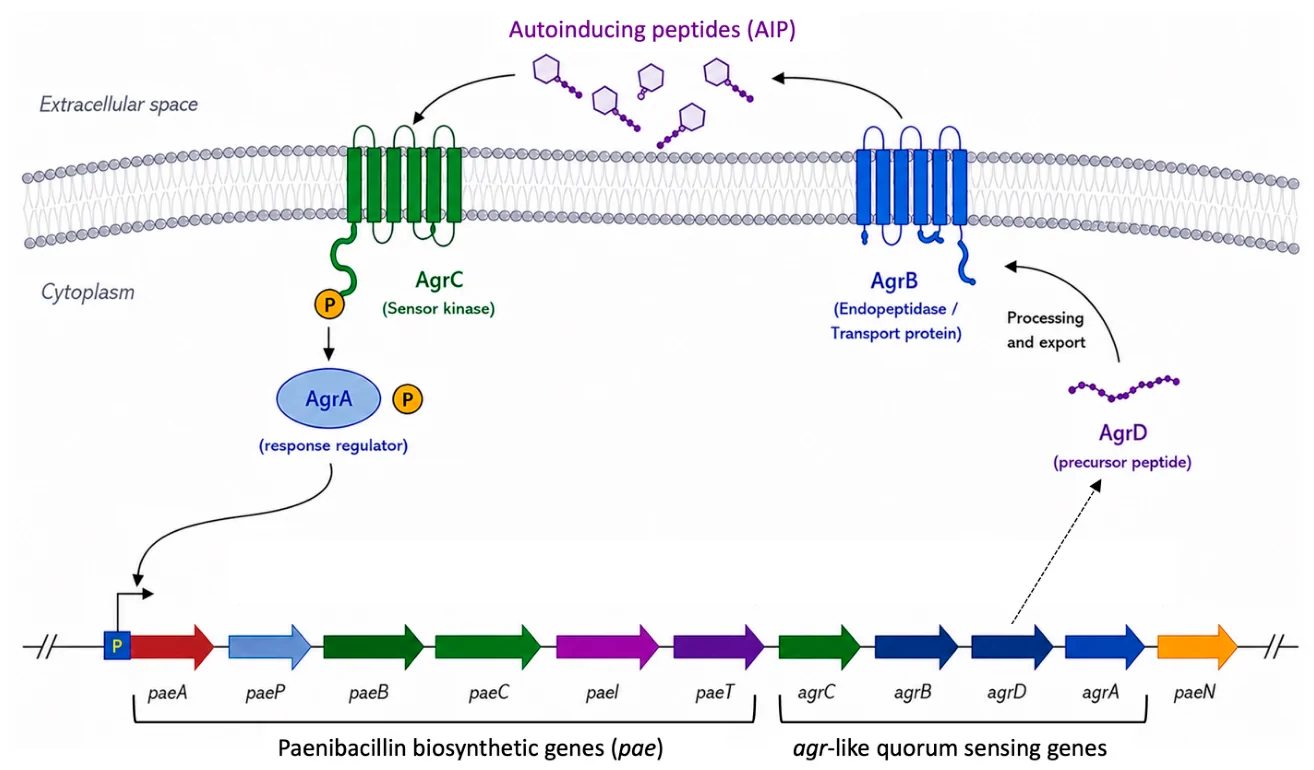
Proposed model for regulating paenibacillin biosynthesis by the Agr-like quorum sensing (QS) system in *Paenibacillus polymyxa* [21,23]. The AgrD gene is transcribed (dotted arrow), processed, and transported by AgrB, an endopeptidase and transport protein, to produce the autoinducing peptides (AIPs) [20,23]. Extracellular accumulation of AIP leads to its binding to the sensor kinase AgrC, triggering autophosphorylation and subsequent transfer of the phosphate to the response regulator AgrA. Phosphorylated AgrA is hypothesized to bind to the promoter (P) upstream of paeA in the paenibacillin biosynthetic gene cluster (pae) and influence its expression. Experimentally, the proposed regulatory connection has not been fully validated.

The paenibacillin biosynthesis gene cluster (BGC) of *P. polymyxa* OSY-DF has four *agr*-like genes, but their functional relationship to paenibacillin biosynthesis has not been well established [23]. The paenibacillin BGC consists of 11 genes including seven genes (*paeA, paeB, paeC, paeI, paeN, paeP*, and *paeT*) predicted to encode for the precursor peptide, post-translational modifying enzymes, and immunity and transport proteins. These are followed by four genes (*agrA, agrB, agrC*, and *agrD*) predicted to be part of an Agr-like quorum sensing system. Based on the presence of the Agr-like QS genes, within the paenibacillin biosynthetic gene cluster, we hypothesize that the putative regulatory system contributes to paenibacillin biosynthesis by *P. polymyxa* OSY-DF (Figure 1). Based on the established mechanisms of Agr QS systems in other Gram-positive bacteria, the proposed model suggests that accumulation of the AgrD-derived autoinducing peptide (AIP) activates the AgrC-AgrA two-component system, and the phosphorylated AgrA may subsequently influence the expression of the paenibacillin biosynthetic genes. However, the proposed regulatory relationship has yet to be validated.

The current study was initiated to determine whether Agr-like and AI-2 QS systems are linked to paenibacillin biosynthesis in *P. polymyxa* OSY-DF. To examine whether expression of the putative Agr-like locus is temporally associated with paenibacillin biosynthesis, we compared the transcriptional patterns of both biosynthetic and putative regulatory genes throughout the growth cycle of *P. polymyxa* OSY-DF. The analysis may reveal transcription patterns that are potentially consistent with gene regulation. Additionally, in separate experiments we investigated whether *P. polymyxa* OSY-DF and OSY-EC produce detectable AI-2 activity and whether perturbation of this activity is associated with changes in paenibacillin production. Exploring the Agr-like and AI-2 QS systems in *P. polymyxa* OSY-DF could provide valuable insights into factors associated with the regulation of paenibacillin biosynthesis. These findings could help identify potential targets for future genetic manipulation that can boost paenibacillin production at a relatively large scale. Increased production would facilitate future studies evaluating the safety, efficacy, and stability of paenibacillin in complex food matrices, ultimately supporting its development as a natural food biopreservative.

## 2. Results

### 2.1. Paenibacillin Production During Growth of Paenibacillus polymyxa OSY-DF

**Figure 2:**
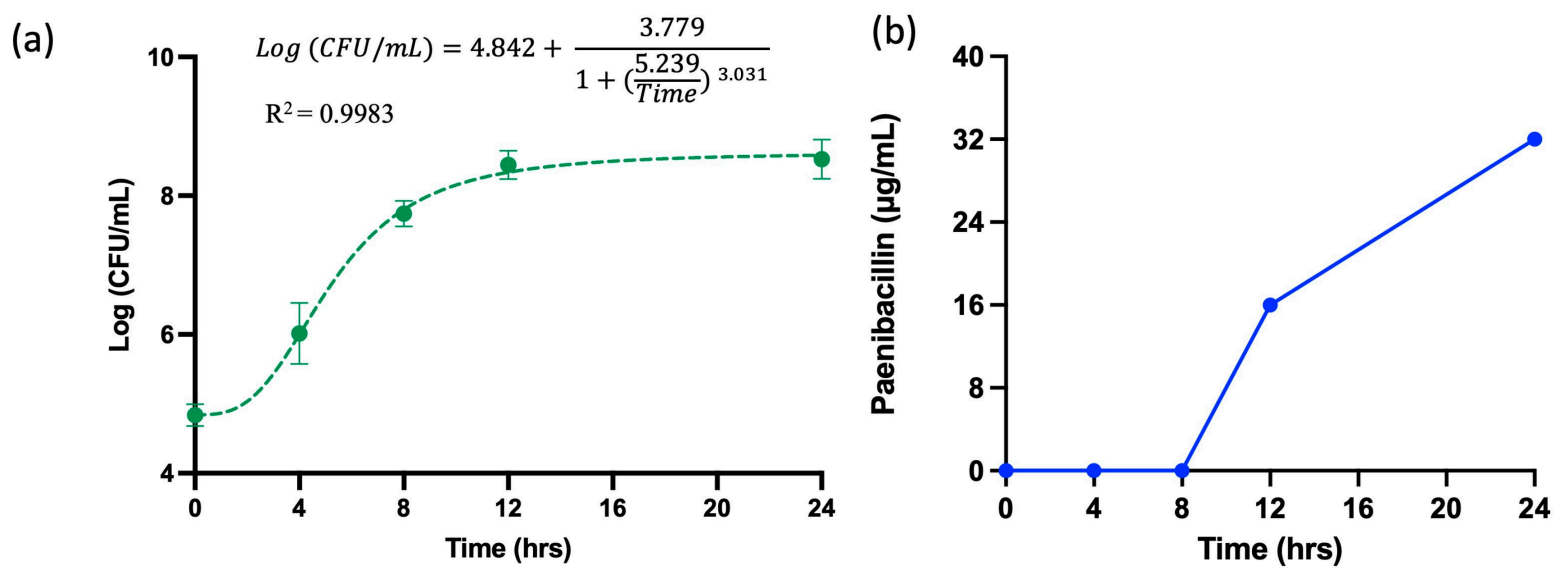
*Paenibacillus polymyxa* OSY-DF growth (panel (**a**)) and paenibacillin concentration determined semi-quantitatively (panel (**b**)) during 24 h of incubation in tryptic soy broth, supplemented with yeast extract, at 30 °C and shaking at 200 rpm. The growth was measured as changes in population counts (log_10_ CFU/mL) and paenibacillin antimicrobial activity was semi-quantitatively determined using an MIC-based method against *Listeria innocua* and presented as paenibacillin concentration (µg/mL). Each data point represents the mean of three biological replicates, and error bars represent ± SD.

Preliminary experiments were conducted to track the growth of *P. polymyxa* OSY-DF and paenibacillin production during 36 h runs (Figures S1 and S2). The results show that the maximum growth of *P. polymyxa* OSY-DF was attained after 12 h of incubation, whereas maximum paenibacillin antimicrobial activity was observed after 24 h of incubation. Previous characterization of *P. polymyxa* OSY-DF established paenibacillin as the antimicrobial compound responsible for its activity against *L. innocua* [7]; therefore, antimicrobial activity against *L. innocua* was used as an indicator of paenibacillin antimicrobial activity in this study. Guided by the results observed over the 36 h run, new growth and antimicrobial production experiments were completed, and transcriptional analysis was monitored during the first 24 h of growth. In these refined experiments, the bacterium completed its growth exponential phase during the first 12 h of incubation, with the growth inflection point (i.e., transition from the acceleration to the deceleration growth rate) observed at ~4 h of incubation (Figure 2a). Paenibacillin antimicrobial activity was first observed at STP12 (Figure 2b), with an estimated concentration of 16 µg/mL. The first detectable paenibacillin production aligns with the high cell density (>8 log_10_ CFU/mL) observed as the growth transitions to the stationary phase (Figure 2). Compared with STP12, higher paenibacillin concentration (estimated to be 32 µg/mL) was observed at STP24 (Figure 2b). No antimicrobial activity was detected at STP0, STP4, or STP8. These results show the association of paenibacillin production with the stationary phase of the *P. polymyxa* OSY-DF growth cycle, which is a typical trend for secondary metabolites production [24].

### 2.2. Time-Resolved Transcriptional Profiling of Paenibacillin Biosynthesis and Agr-like Quorum Sensing Genes

**Figure 3:**
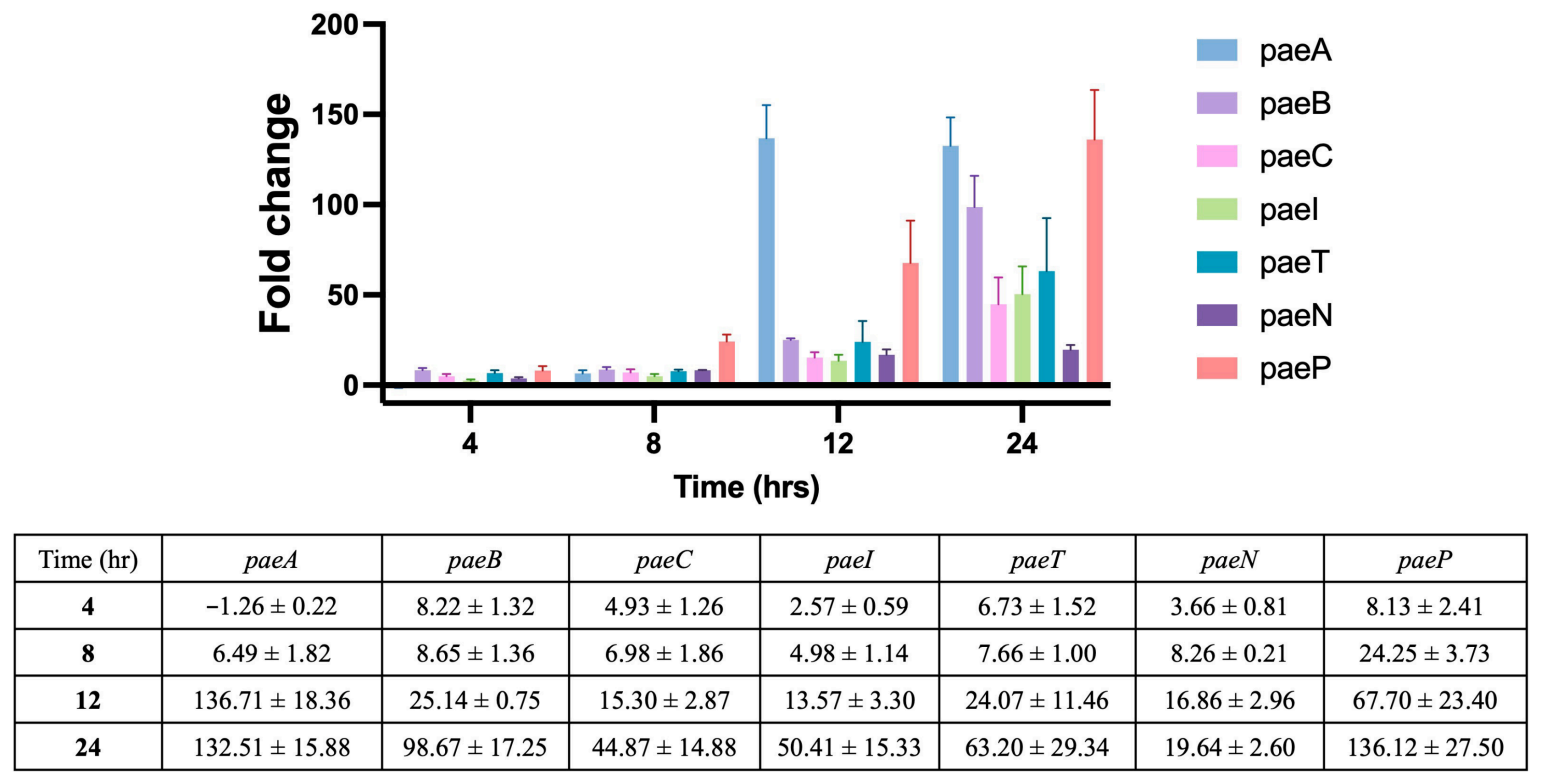
Transcription of paenibacillin biosynthetic (*pae*) genes in *Paenibacillus polymyxa* OSY-DF over 24 h of incubation. Transcription levels were normalized to the 16S rRNA housekeeping gene, and relative expression was calculated by normalizing each gene to its expression at sampling time point 0. Data are shown as the mean ± SD of three independent biological experiments. Technical replicates (*n* = 4) were averaged for each biological replicate. Genes represented in the graph are encoding the following products: PaeA: paenibacillin prepropeptide; PaeB: lantibiotic dehydratase; PaeC: lantibiotic cyclase; PaeP: peptidase; PaeI: immunity protein; PaeN: putative acetylase; PaeT: transporter protein.

**Figure 4:**
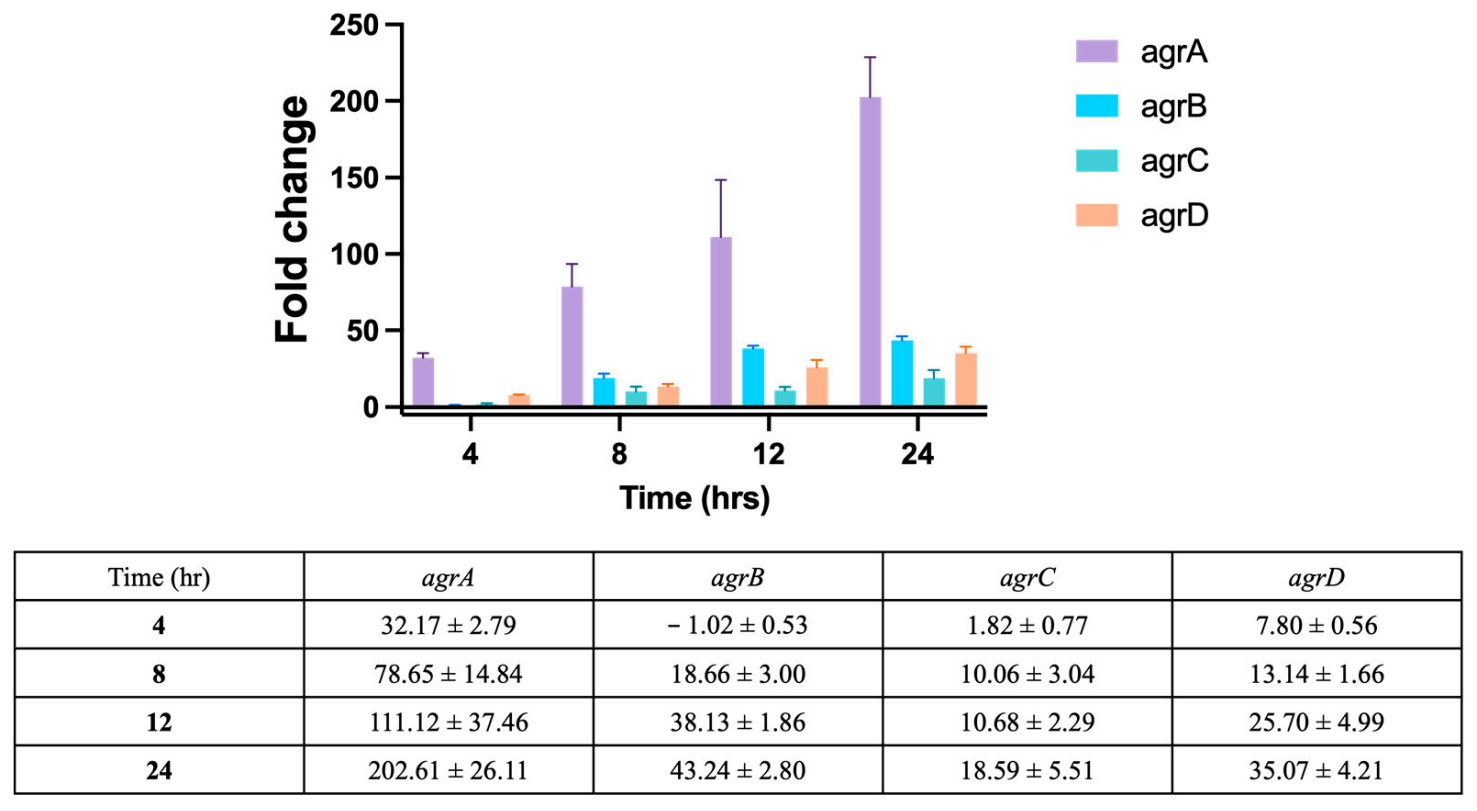
Transcription of accessory gene regulator (Agr)-like quorum sensing genes in *Paenibacillus polymyxa* OSY-DF over 24 h of incubation. Transcription levels were normalized to the 16S rRNA housekeeping gene, and relative expression was calculated by normalizing each gene to its expression at sampling time point 0. Data are shown as the mean ± SD of three independent biological experiments. Technical replicates (*n* = 4) were averaged for each biological replicate. Genes represented in the graph are encoding the following products: AgrA: Response regulator; AgrB: Autoinducing peptide processing and export protein; AgrC: Histidine kinase; AgrD: Autoinducing peptide (AIP).

The transcription of paenibacillin seven biosynthetic genes (*paeA, paeB, paeC, paeI, paeN, paeP*, and *paeT*) and its four putative regulatory genes (*agrA, agrB, agrC*, and *agrD*) were quantified during the growth cycle of the producer, *P. polymyxa* OSY-DF, using RT-qPCR. As indicated in the method’s section, the RT-qPCR values were normalized against a house-keeping gene (16S rRNA gene) and against the transcription values observed at 0 h of incubation. Comparing biosynthesis and transcription patterns (Figure 3 and Figure 4) reveals potential correlation. The gene encoding the pre-pro-peptide (*paeA*) was initially downregulated at STP4, exhibiting a 1.26-fold decrease (Figure 3); however, the transcription subsequently increased steadily, with the highest (136-fold increase) observed at STP12. For the remaining six genes (*paeB, paeC, paeI, paeN, paeP*, and *paeT*), a transcription pattern similar to that of *paeA* was observed. Generally, an upregulation trend was observed as time increased. The overall transcriptional analysis revealed a substantial increase in the transcription of the seven paenibacillin biosynthetic genes after 24 h of incubating *P. polymyxa* OSY-DF under optimum growth conditions and the peak of transcription appearing at the 12 and 24 h sampling timepoints (STP12 and STP24).

In the case of the four putative Agr-like quorum sensing genes (*agrA, agrB, agrC*, and *agrD*), a similar upregulation with incubation time was observed (Figure 4). With the exception of *agrB*, which showed initial downregulation at STP4 (1.02-fold decrease), all other genes (*agrA, agrC*, and *agrD*) showed upregulation at STP4. A steady, almost linear, increase in upregulation of the Agr-like genes (*agrABCD*) was observed throughout the 24 h of incubation. At STP8 and STP12, these genes exhibited between a 10.06- and 78.65-fold increase, and between a 10.68- and 111.12- fold increase, respectively. The highest upregulation of all Agr-like genes (*agrABCD*) was observed at STP24, showing between a 18.59- and 202.59-fold increase (Figure 4). When the transcription patterns of the biosynthetic (Figure 3) and the putative regulatory (Figure 4) genes were compared, upregulation of several Agr-like genes was detected earlier than the strongest induction of the paenibacillin biosynthetic genes.

**Figure 5:**
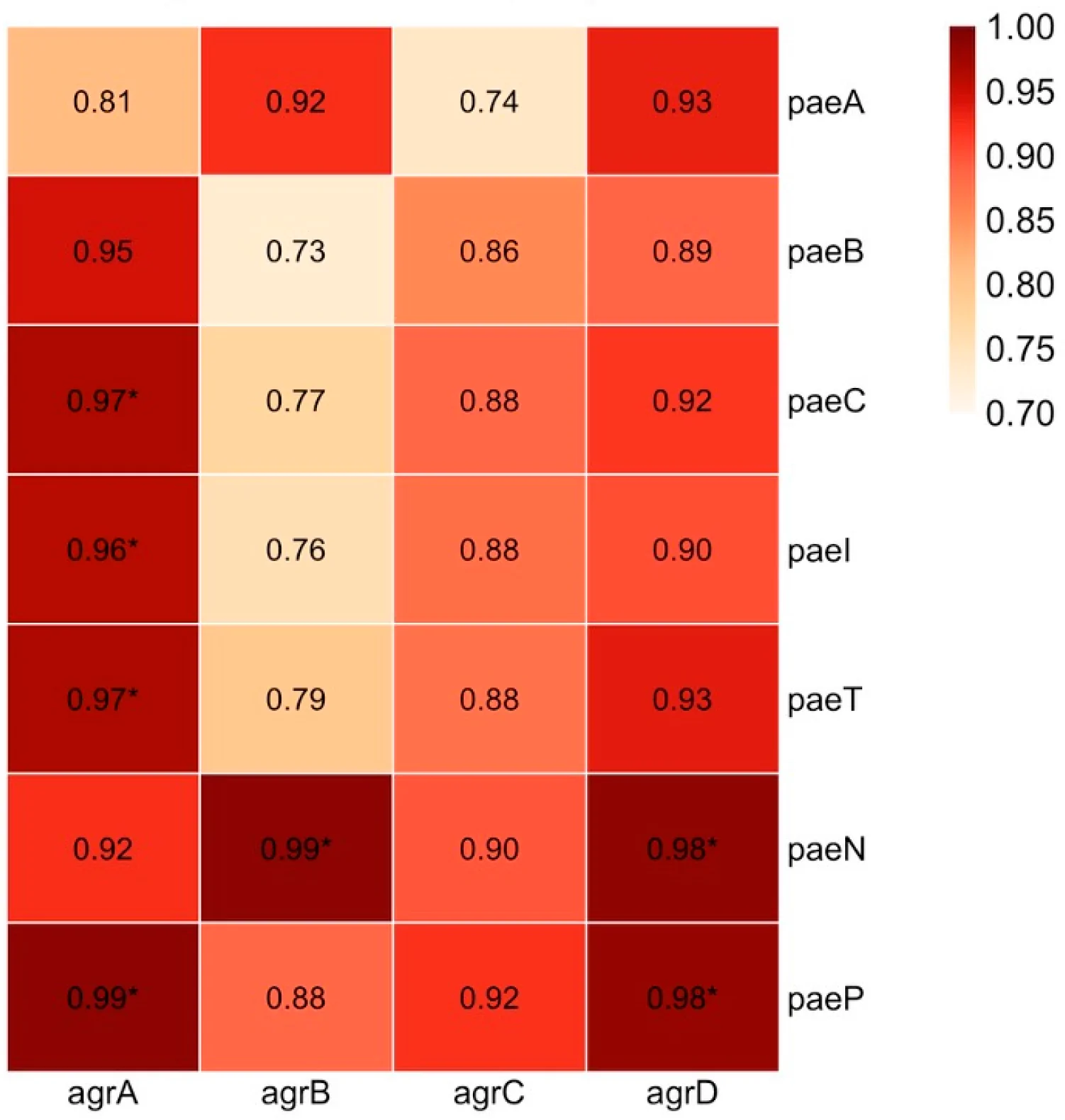
Pearson correlation analysis of temporal expression profiles of the Agr-like quorum sensing genes and the paenibacillin biosynthetic (*pae*) genes. Correlation coefficients (*r*) were calculated from the mean relative gene expression values measured at 4, 8, 12, and 24 h. Pearson correlation coefficients are shown within each cell. The color scale was adjusted to 0.70–1.00 to enhance visualization of differences among positive correlations. The asterisk (*) indicates significantly correlated gene pairs (adjusted to *p <* 0.05) after Benjamini–Hochberg false discovery rate correction.

Pearson correlation analysis was performed to evaluate the transcriptional temporal relationship between the Agr-like genes and the paenibacillin biosynthetic (Pae) genes (Figure 5). Positive correlations were observed between all Agr-like and Pae gene pairs, with Pearson correlation coefficients ranging from 0.73 to 0.99. The strongest positive correlations were observed between *agrB* and *paeN* (r = 0.99), *agrA* and *paeP* (r = 0.99), and *agrD* with *paeN* and *paeP* (r = 0.98). Overall, positive temporal correlations were observed between Agr-like genes and those of the paenibacillin biosynthetic gene cluster throughout the growth curve.

### 2.3. Paenibacillus polymyxa Strains Produce Detectable AI-2 Quorum Sensing Signals

**Figure 6:**
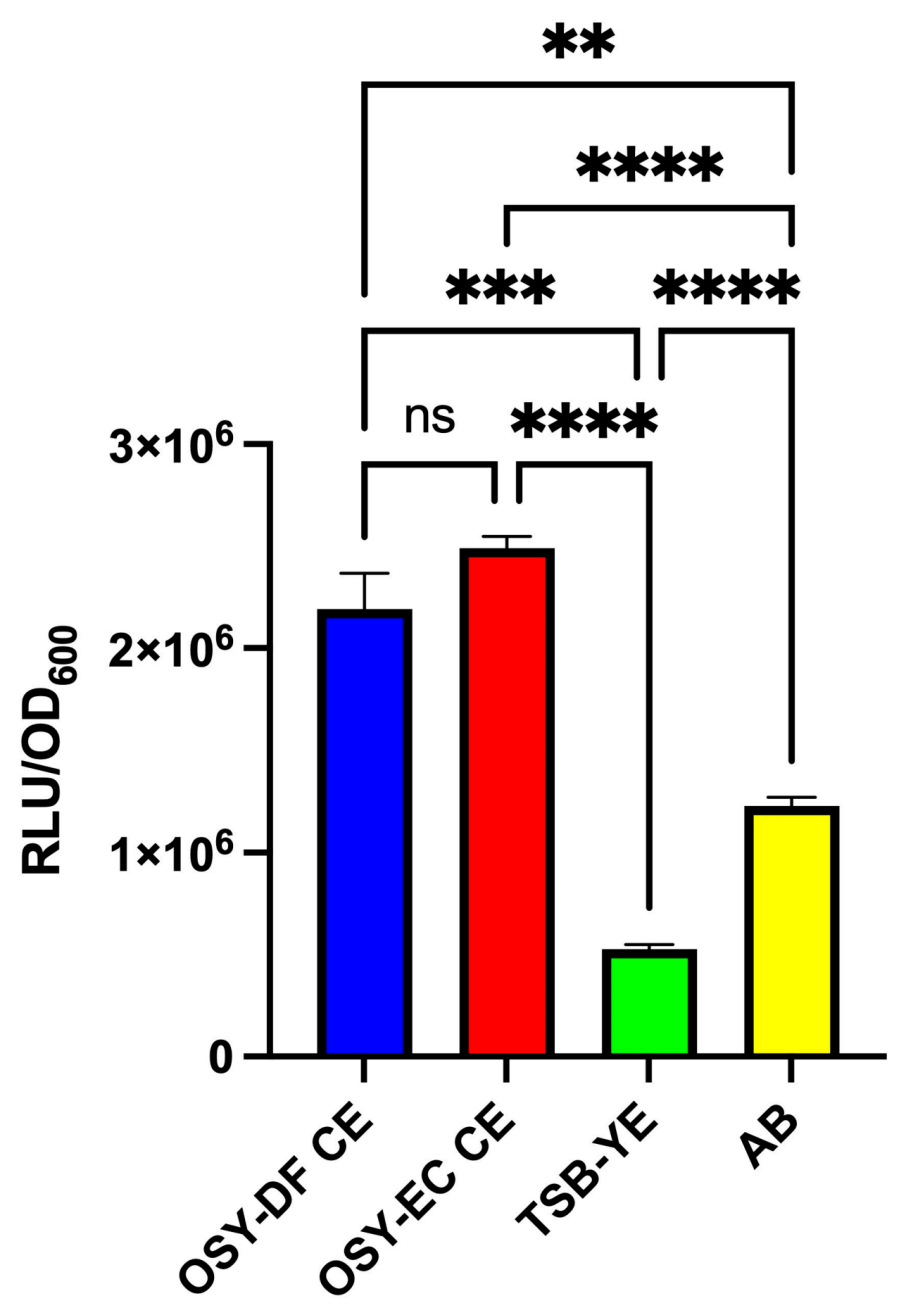
Detection of the antimicrobial producers’ quorum sensing AI-2 signals, measured as relative luminescence units (RLUs)/OD600, reported by *Vibrio harveyi*, which was treated with cell-free extract (CE) from *Paenibacillus polymyxa* strains (OSY-DF or OSY-EC) after 24 h of incubation. The controls are the media, tryptic soy broth with yeast extract (TSB-YE), and autoinducer bioassay broth (AB), inoculated with *V. harveyi*. Data are presented as the means of four replicates ± SD. Asterisks denote significant differences: (**), *p* < 0.01; (***), *p* < 0.001: (****), *p* < 0.0001. “ns” denotes non-significant (*p* > 0.05).

In a separate set of experiments from the Agr-associated transcriptional studies, AI-2-associated activity was investigated in *P. polymyxa* OSY-DF and OSY-EC, a ribosomally engineered derivative of the wild-type OSY-DF strain [12]. Treatment of *V. harveyi* with cell-free filtrates of *P. polymyxa* strains OSY-DF and OSY-EC produced significantly higher luminescence/OD signal (*p* < 0.05, Figure 6) compared with the untreated control, in which the cell-free filtrates (extracts) were replaced with equivalent volumes of TSB-YE or AB medium (Figure 6). However, the responses of *V. harveyi* to the treatment with filtrates from *P. polymyxa* OSY-DF and *P. polymyxa* OSY-EC were not significantly different (*p* > 0.05). The increase in luminescence produced by *V. harveyi* when treated with the cell-free filtrate from the *P. polymyxa* strains indicates detectable AI-2 signaling activity in both *P. polymyxa* strains OSY-DF and OSY-EC. In addition, genes associated with LuxS-dependent AI-2 precursor formation, including *luxS*, were detected in the genomes of the two *P. polymyxa* strains (Table S1).

### 2.4. D-Ribose Decreased AI-2 Signals Detected in Cultures of P. polymyxa Strains but Did Not Reduce Paenibacillin Activity

**Figure 7:**
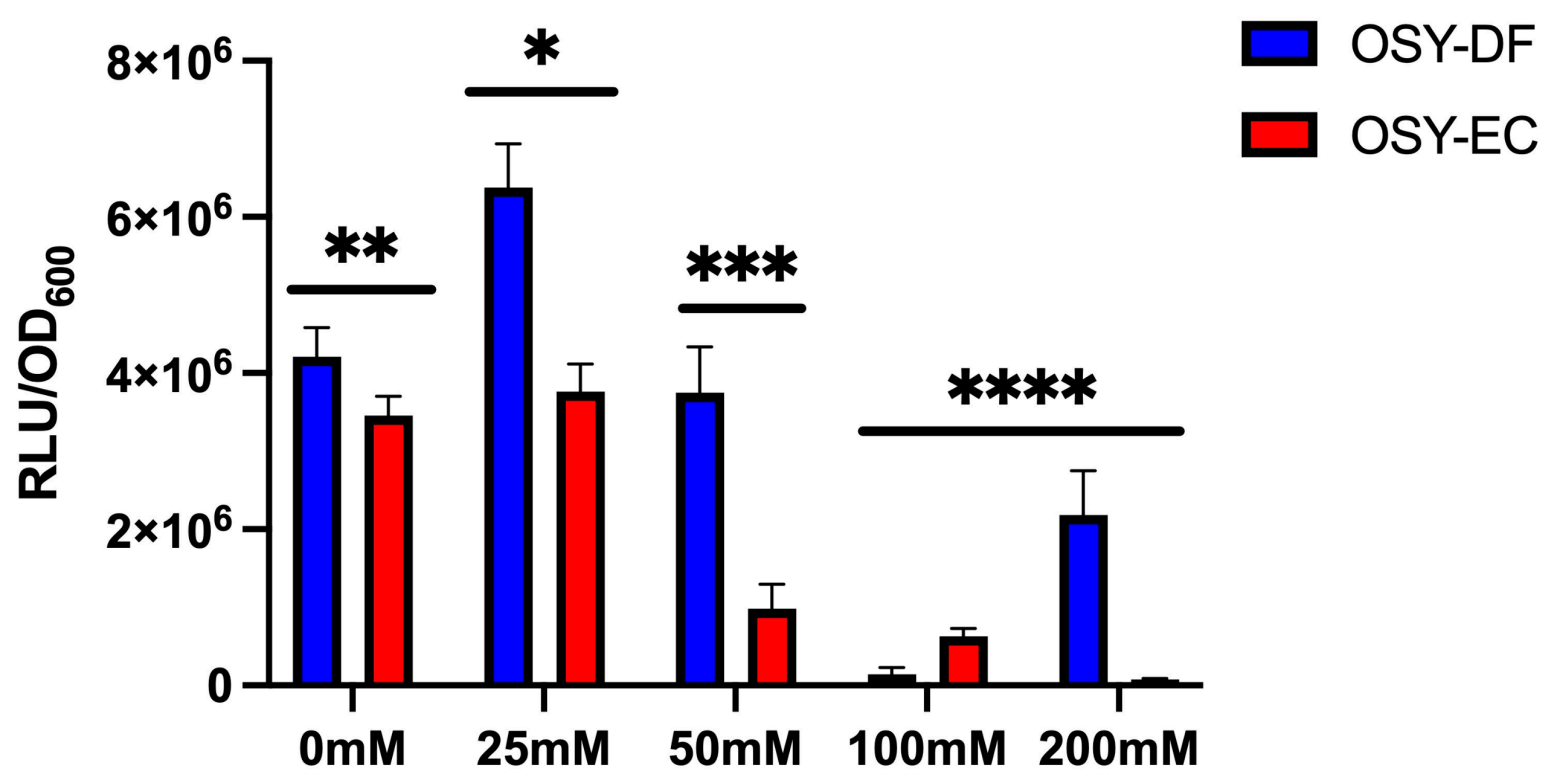
AI-2 signaling activity detected in *Paenibacillus polymyxa* OSY-DF and OSY-EC following D-ribose treatment. AI-2 signaling activity was measured as relative luminescence units (RLUs)/OD600, reported by *Vibrio harveyi*, which was treated with cell-free extract (CE) from *P. polymyxa* strains (OSY-DF or OSY-EC) grown in varying concentrations of an AI-2 inhibitor (D-ribose) after 24 h of incubation. Data are presented as the means of four replicates ± SD. Asterisks denote significant differences: (*), *p* < 0.05; (**), *p* < 0.01; (***), *p* < 0.001: (****), *p* < 0.0001.

As the concentration of D-ribose in *P. polymyxa* cultures increased, the response of *V. harveyi* to the treatment with *P. polymyxa* cell-free filtrates varied depending on the antimicrobial-producing strains (i.e., *P. polymyxa* OSY-DF vs. OSY-EC) (Figure 7). The response, measured as luminescence/OD value, is indicative of the strength of the AI-2 signal produced by *P. polymyxa* and detected by *V. harveyi* [25]. For the AI-2 signals detected in the culture of the mutant, *P. polymyxa* OSY-EC decreased as D-ribose concentration exceeded 25 mM. On the contrary, the AI-2 signal detected in the culture of the paenibacillin-producing wild type (*P. polymyxa* OSY-DF) increased considerably at 25 mM D-ribose, compared with the untreated control, and then decreased at the higher concentrations of AI-2 inhibitor, with signal rebound at 200 mM D-ribose. The highest concentrations of D-ribose (100 and 200 mM) significantly decreased the AI-2 signals produced by both paenibacillin-producer strains (Figure 7), compared with the untreated control.

**Figure 8:**
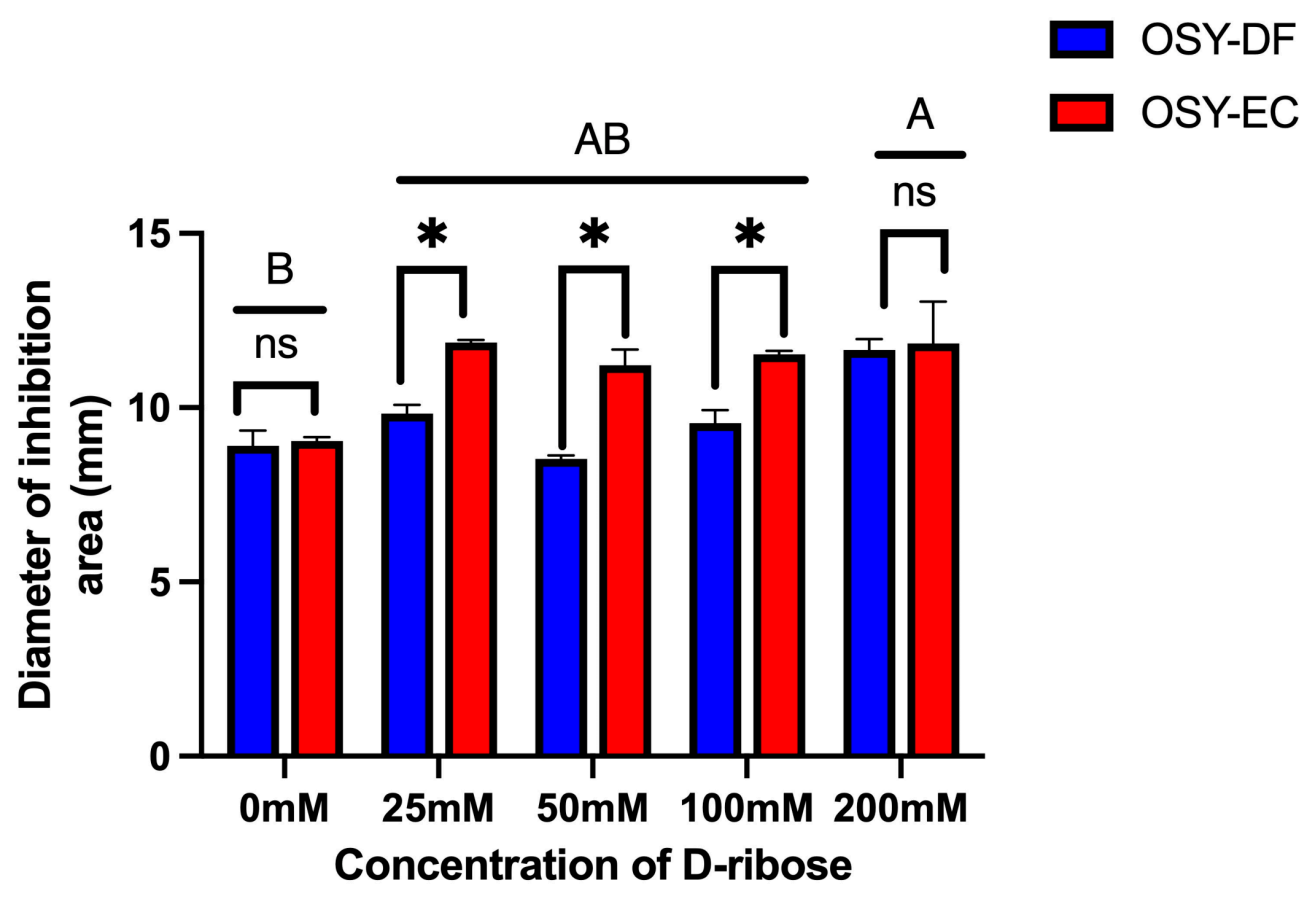
Antimicrobial activity, measured as diameters of inhibition areas expressed by the indicator *Listeria innocua* during agar diffusion bioassay, for cell-free extract (CE) from *Paenibacillus polymyxa* strains (OSY-DF or OSY-EC), that were treated with increasing concentrations of D-ribose. Data are presented as the means of four replicates ± SD. Asterisks indicate significantly different strains (*p* < 0.05), and “ns” indicates differences were not significant. Different letters (A and B) indicate significant differences among D-ribose concentrations (*p <* 0.05); treatments sharing at least one letter (AB) are not significantly different.

In addition to measuring AI-2 signal, paenibacillin antimicrobial activity in the cell-free filtrates from D-ribose-treated *P. polymyxa* strains was determined (Figure 8). *L. innocua* was used as the indicator strain to detect the anti-Gram-positive effect of paenibacillin. The decrease in AI-2 activity resulting from the increased concentrations of D-ribose was not accompanied by a corresponding reduction in paenibacillin antimicrobial activity (Figure 8). Antimicrobial activity, measured as the diameter of the inhibition areas of *L. innocua*, increased with increasing concentrations of D-ribose. The antimicrobial activity of both OSY-DF and OSY-EC strains treated with 200 mM D-ribose showed significantly higher (*p* < 0.05) antimicrobial activity than the untreated (0 mM D-ribose) group (Figure 8). The diameters of the inhibition areas resulting from the cell-free filtrates were greater (*p* < 0.05) in extracts of OSY-EC strain cultures, compared with those of OSY-DF strain cultures, for all ribose concentrations except the 200 mM concentration (Figure 8). In conclusion, although AI-2 activity decreased with increased D-ribose concentration, paenibacillin antimicrobial activity against *L. innocua* did not decrease.

Additionally, to distinguish between the potential confounding effects of high sugar concentrations from those associated with AI-2 inhibition, additional experiments were conducted using 0, 100, and 200 mM D-ribose and 200 mM sucrose as a carbohydrate control. Higher concentrations of D-ribose and 200 mM sucrose resulted in increased growth of both OSY-DF and OSY-EC (Figure S3) as compared with the untreated control (0 mM D-ribose). Detectable AI-2 signaling activity decreased at higher D-ribose concentrations as observed earlier (Figure 7), whereas 200 mM sucrose did not result in a corresponding decrease in AI-2 signaling activity (Figure S4). Antimicrobial activity increased in the presence of both higher D-ribose concentrations and 200 mM sucrose (Figure S5). These results suggest that the slight increase in antimicrobial activity at higher sugar concentrations may be associated with increased bacterial growth rather than AI-2 inhibition.

Additionally, to determine whether D-ribose or sucrose directly affected the reporter response, *V. harveyi* BB170 was also treated with 10% of TSB-YE containing 0, 25, 50, 100, or 200 mM D-ribose or 200 mM sucrose without *P. polymyxa* inoculation. No significant differences in the *V. harveyi* BB170 reporter response were observed among these treatments (Figure S6), suggesting that the tested concentrations of D-ribose and sucrose did not significantly interfere with the reporter assay.

## 3. Discussion

The control of foodborne pathogens and spoilage microorganisms is of utmost importance especially given the rise in antimicrobial resistance [26]. The use of novel, effective, and natural antimicrobials, such as paenibacillin, potentially offers a promising and a clean-label friendly solution for food preservation and safety [6]. However, to test the potential use of paenibacillin as a natural food preservative, large-scale production of paenibacillin is required, which is limited by the low natural production of the antimicrobial peptide by the producer strain [11]. Before paenibacillin, and similar novel antimicrobials, can be used in industrial applications, research is needed to support regulatory approval, including evaluation of its safety, efficacy, and stability in complex food matrices. Genetic engineering can be a powerful tool to increase antimicrobial production. However, to effectively use this technology, it is crucial to understand the regulation of biosynthesis of target antimicrobials [27]. Revealing such regulatory mechanisms would contribute to a broader understanding of the biosynthesis of lantibiotics and other secondary metabolites. Quorum sensing systems regulate various secondary metabolites, including some associated with antimicrobials production [15]. For example, the antimicrobial producers, *Lactobacillus plantarum* and *Lactobacillus gasseri*, use quorum sensing systems to regulate bacteriocin production [28]. Exploring and modulating quorum sensing pathways in antimicrobial producers may be a viable path to enhance the biosynthesis of these antimicrobials.

In the current study, we aimed to investigate the potential associations between paenibacillin biosynthesis and two different quorum sensing systems: the Agr-based and AI-2-based, systems. *P. polymyxa* strains carry a set of presumptive regulatory genes called the accessory gene regulators, *agr* [23]. Such regulators are known to contribute to the expression of *Staphylococcus aureus* virulence [29]. The predicted Agr-like genes in *P. polymyxa* OSY-DF are located within the paenibacillin BGC [23]. Although their co-location does not confirm a regulatory relationship, it may hint to the potential involvement of the Agr-like genes in paenibacillin biosynthesis. The Agr-based regulation is typically activated once a high population density is reached, most often during the late exponential to early stationary phases [17,20]. This timing coincides with the production of bacteriocins, which are also commonly synthesized during the late growth phases [24]. Quorum sensing-mediated regulation of bacteriocins has been observed in other Gram-positive species (e.g., *Lactococcus lactis*), particularly at high cell densities in the late exponential to early stationary phase [30,31]. Similarly, the timing of gene transcription within the paenibacillin BGC and antimicrobial production may suggest a potential association between the Agr-like quorum sensing system and paenibacillin biosynthesis. However, functional genetic studies are needed to validate the hypothesized role of the Agr-like system in paenibacillin regulation.

For the transcriptional analysis, the parental OSY-DF strain was selected to characterize temporal expression of the native paenibacillin BGC and its associated Agr-like locus without potential confounding effects from the altered phenotype of the spontaneous OSY-EC mutant. The transcriptional analysis of the paenibacillin BGC and expression of the antimicrobial agent coincided with the maximum growth (STP24; Figure 2, Figure 3 and Figure 4). Additionally, the Agr-like genes showed a transcriptional profile that preceded that of the paenibacillin biosynthetic genes, with both reaching their maxima at STP24 (Figure 3 and Figure 4). The transcriptional delay of the seven biosynthesis genes suggests that sufficient autoinducing peptide (AIP) signals, produced by the Agr-like QS system, may be needed to reach a threshold value that triggered the transcription of the rest of the paenibacillin BGC. Additionally, paenibacillin biosynthesis and secretion, as determined by the agar diffusion bioassay, was first detected after 12 h of incubation and increased during the remainder of incubation. The staggered transcription of the Agr-like and paenibacillin biosynthetic (*pae*) genes may suggest a potential regulatory linkage between these two systems. The correlation analysis further demonstrated that the Agr-like genes and the paenibacillin biosynthetic genes exhibited similar temporal transcription patterns throughout 24 h, with strong positive correlations observed across all gene pairs. These findings support the possibility that an Agr-like QS system is associated with paenibacillin biosynthesis. However, because correlation does not establish causation, additional studies, such as targeted genetic knockouts or complementation analyses, are required to determine whether the Agr-like QS system directly regulates paenibacillin production.

In some bacteriocin BGCs, quorum sensing genes are located adjacent to or embedded within the cluster, where they function as dedicated regulatory elements for antimicrobial production [30,32]. The colocalization of Agr genes within the paenibacillin BGC, suggests a potential association with paenibacillin biosynthesis; however, further functional studies are required to validate this hypothesis and establish a regulatory relationship. Although *P. polymyxa* ATCC 842 was not found to produce paenibacillin (unpublished data), other researchers harvested the antimicrobial agent from a large bioreaction batch (37). When these researchers developed a mutant of *P. polymyxa* ATCC842 with an inactivated Agr-like system, they observed significant downregulation of genes in the paenibacillin BGC as well as lower detection of paenibacillin by chromatographic analysis. These findings further support the hypothetical role of Agr-like QS systems in regulating paenibacillin biosynthesis in *P. polymyxa* OSY-DF.

Based on the study findings, both paenibacillin-producing strains, *P. polymyxa* OSY-DF and OSY-EC, produce detectable AI-2 signals (Figure 6). This aligns with their genetic profile since both strains contain *luxS* that encodes for AI-2 synthase, which is required for synthesizing AI-2 molecules (Table S1). In a previous study, the presence of *luxS* and its positive impact on biofilm production through AI-2 signaling molecules were reported in *P. polymyxa* HY96-2 [33]. On the contrary, other *P. polymyxa* strains that contain *luxS* did not produce detectable AI-2 signals [34,35]. These findings suggest that although *luxS*, or its homologs, were detected in *P. polymyxa* strains, the expression of AI-2 is strain-dependent.

Contrary to our prediction, inhibition of AI-2 signaling by D-ribose did not decrease paenibacillin activity (Figure 8). These findings suggest that AI-2 signaling may not significantly contribute to paenibacillin biosynthesis under the tested conditions; further studies are required to confirm this relationship. At high concentrations (100–200 mM) of D-ribose (Figure 8), paenibacillin activity increased in both OSY-DF and OSY-EC strains (Figure 8). It is plausible that the increase is related to alterations in central carbon metabolism, which would indirectly influence antimicrobial production in *P. polymyxa*. Additional experiments conducted to evaluate the potential confounding effects of high sugar concentrations showed that higher D-ribose concentrations also resulted in increased bacterial growth (Figure S3) and antimicrobial activity (Figure S5). Similarly, 200 mM sucrose increased bacterial growth and antimicrobial activity but did not decrease detectable AI-2 activity (Figures S3–S5). These findings suggest that the increased antimicrobial activity observed at high sugar concentrations may be associated with increased bacterial growth and/or changes in carbon metabolism rather than inhibition of AI-2 signaling. Further studies are needed to determine the mechanisms underlying these effects.

In Gram-negative bacteria, LuxP/LuxQ or Lsr ABC transporter systems are used to sense and internalize AI-2 signals, and in some Gram-positive bacteria, ribose-binding protein (RbsB) is used to uptake AI-2 signals [19,36]. Consequently, a transcriptional regulator like LuxR is activated, which is responsible for the upregulation or downregulation of target genes [19]. The genomes of the two *P. polymyxa* strains contain both *rbsB* to internalize AI-2 signals and multiple copies of *luxR* (Table S1), which may transcriptionally regulate target genes. However, the presence of these genes does not necessarily demonstrate their functional involvement in regulating paenibacillin biosynthesis. Despite these observations, paenibacillin production did not decrease when AI-2 signals were inhibited. Therefore, the findings of the present study did not prove the role for AI-2 signaling in regulating paenibacillin biosynthesis under the experimental conditions evaluated. Considering these *P. polymyxa* strains produce detectable AI-2 signals and carry multiple copies of *luxR*, the strains may utilize AI-2 signaling to regulate other functions such as biofilm formation or production of other secondary metabolites; however, this remains to be experimentally confirmed.

## 4. Limitations and Future Directions

Despite the insights gained from this study, several limitations need to be considered. Although our findings suggest a potential association between the Agr-like quorum sensing system and paenibacillin biosynthesis, functional validation through gene knockout and complementation is needed to establish a regulatory relationship. The construction of Δ*agr* mutants has been under investigation; however, the recalcitrant nature of *P. polymyxa* has made genetic manipulation technically challenging. Genome mining of *P. polymyxa* OSY-DF identified multiple putative restriction-modification (R-M) system genes, including genes encoding restriction endonucleases and DNA methyltransferases (Table S2). The presence of these putative R-M systems may contribute to the difficulty in introducing foreign DNA into *P. polymyxa* OSY-DF. The presence of R-M systems restricting exogenous DNA has been reported in other *P. polymyxa* strains [37].

Future work may include direct quantification of AIP, AI-2 signaling molecules, and paenibacillin production using LC-MS based analytical methods, together with functional genetic studies, to further elucidate the role of quorum sensing systems in paenibacillin biosynthesis. It is also possible that additional regulatory networks may contribute to paenibacillin biosynthesis. These cannot be excluded based on the current data, and further studies are needed to investigate these potential regulatory mechanisms.

## 5. Materials and Methods

### 5.1. Bacterial Strains, Growth Media, and Culturing Conditions

All strains used in this study were obtained from the culture collection of Food Microbiology Laboratory at The Ohio State University (Columbus, OH, USA). *P. polymyxa* OSY-DF was originally isolated from kimchi and identified in our laboratory [7]. The genome of OSY-DF was subsequently sequenced and assembled in our laboratory and is publicly available in NCBI under genome assembly accession number GCA_000265445.1. The paenibacillin-producing wild-type strain, *P. polymyxa* OSY-DF [7], and its mutant, *P. polymyxa* OSY-EC [12], were used to evaluate the potential relationship between AI-2 QS system and paenibacillin production. However, for the transcriptional analysis of paenibacillin biosynthetic genes and Agr-like QS genes, only the wild-type strain was used. The mutant, *P. polymyxa* OSY-EC, was excluded considering it was generated through sequential antibiotic screening [12], which may have caused uncharacterized genetic alterations directly affecting the transcription of paenibacillin biosynthesis genes. The paenibacillin-producing strains were grown in tryptic soy broth (Becton Dickinson, Sparks, MD, USA) supplemented with 0.6% yeast extract (TSB-YE) at 30 °C with agitation at 200 rpm for 24 h. When bacterial populations were counted, tryptic soy agar (TSA) was used. The anti-Gram-positive antimicrobial indicator strain, *Listeria innocua* ATCC 33090, was grown in lysogeny broth (LB; Fisher Scientific, Fair Lawn, NJ, USA) at 37 °C for 12 h. A luminescence sensor strain, *Vibrio harveyi* BB170, was grown in an autoinducer bioassay (AB) medium [25], which contained the following ingredients per liter: NaCl, 17.5 g; MgSO_4_, 12.3 g; casamino acid, 2 g; 1 M K_3_PO_4_, 10 mL; 0.1 M L-arginine, 10 mL; and pure glycerol, 10 mL. The strain was grown in the AB medium by incubation at 30 °C for 24 h.

### 5.2. Paenibacillus polymyxa OSY-DF Growth and Paenibacillin Production Patterns

*P. polymyxa* OSY-DF was grown using a sequential subculturing approach to ensure that cell population was uniformly and actively growing before gene transcription analysis [38]. Briefly, a culture of *P. polymyxa* OSY-DF was grown in 10 mL of fresh TSB-YE medium for 5 h at 30 °C with shaking at 200 rpm. A 1%, inoculum from the 5 h culture was then transferred to fresh TSB-YE medium and incubated for 10 h under the same conditions. To study growth, antimicrobial production, and gene transcription and regulation, a 1% inoculum from the 10 h culture was used to inoculate 100 mL of fresh TSB-YE medium. This test culture was incubated for 24 h at 30 °C while shaking at 200 rpm. Aliquots (1 mL each) of the incubated culture were taken at the following sampling time points (STPs): 0, 4, 8, 12, and 24 h. At each STP, *P. polymyxa* population was counted using the spread-plating method on TSA medium. The growth curves were generated based on a non-linear fitting, using a four-parameter logistic model (Prism 9.4.1; GraphPad Software, San Diego, CA, USA). Cell-free filtrates (extracts) of *P. polymyxa* OSY-DF culture samples were prepared at each STP to monitor the antimicrobial activity of biosynthesized paenibacillin. When needed, cell-free filtrates were stored at −20 °C until further analysis. An antimicrobial agar diffusion bioassay was conducted as described later.

### 5.3. RNA Extraction and cDNA Synthesis

At each STP (0, 4, 8, 12, and 24 h), during growth curve generation, total RNA was extracted from an aliquot (1 mL each) of the *P. polymyxa* OSY-DF culture for gene transcription analysis. The bacterial cells were pelleted by centrifugation and resuspended in 200 µL of saline solution containing 20 µL of lysozyme (80 mg/mL; Sigma-Aldrich, St. Louis, MO, USA) and 20 µL of proteinase K (20 mg/mL; Qiagen, Germantown, MD, USA). The mixture was incubated at 37 °C for 1 h. A commercial RNA extraction kit (RNeasy minikit; Qiagen) was used to extract the RNA, following manufacturer’s instructions with minor modifications. RNA cleanup and the removal of residual DNA were performed using DNase enzyme (Turbo DNase kit; Invitrogen, Vilnius, Lithuania), as described in previously published work [39]. The quantity and quality of RNA was checked using a spectrophotometer (NanoVue; GE Healthcare Life Sciences, Buckinghamshire, UK). A reverse transcription kit (High-Capacity cDNA; Applied Biosystems, Vilnius, Lithuania) was used for the reverse transcription of the purified RNA into cDNA. Reverse transcription was performed using random primers following the manufacturer’s instructions (GeneAmp PCR System 2400; Applied Biosystems, Waltham, MA, USA) in a thermocycler. The cDNA was diluted using 80 µL of sterile deionized water and stored at −20 °C until further analysis.

### 5.4. Real-Time Quantitative PCR (RT-qPCR) for the Gene Transcription Analysis of Paenibacillin Biosynthesis Genes

The primers used for the amplification of the paenibacillin biosynthesis genes in *P. polymyxa* OSY-DF are listed in Table 1 [39]. The 11 genes of the paenibacillin biosynthesis gene cluster (BGC), including seven paenibacillin structural and post-modification genes and four Agr-like genes, were examined during the gene transcription analysis. For the RT-qPCR, 20 µL of reaction mixtures were prepared as follows: 5 µL of template cDNA, 10 µL of SYBR Select PCR master mix (Applied Biosystems), 0.4 µL of each of the 25 µM forward and reverse primers (Millipore Sigma, Danvers, MA, USA), and water to reach the 20 µL volume. An RT-qPCR system (7900HT Fast Real-Time PCR System; Applied Biosystems) was used at the following PCR setting: an initial denaturation step at 95 °C for 7 min, followed by 40 cycles of 95 °C for 15 s and 60 °C for 1 min. The cycle threshold (CT) values were obtained after each run and used for relative gene transcription analysis. The relative gene transcription levels of all genes in the paenibacillin BGC at different STPs were normalized to the 16S rRNA housekeeping gene, and relative expression was compared with gene transcription at 0 h (STP0: the time point immediately after inoculation of the final production culture) using the ΔΔCt method, with results expressed as fold change [40]. For ease of data interpretation and visualization, fold change values that are less than one (downregulation) were transformed to negative values (−1/fold change), whereas positive values indicate upregulation relative to STP0.

### 5.5. Detection of Autoinducer (AI-2) Quorum Sensing Signal in P. polymyxa Strains

Production of AI-2 quorum sensing signal by the paenibacillin-producing strains (*P. polymyxa* OSY-DF and *P. polymyxa* OSY-EC) was determined using a reporter strain, *V. harveyi* BB170, which produces luminescence upon detection of AI-2 signals [25]. This reporter assay provides an indirect assessment of AI-2 biological activity. An overnight culture of *V. harveyi* BB170 was grown in AB medium and diluted 1:1000 in fresh AB medium before use in the assay. *P. polymyxa* OSY-DF and *P. polymyxa* OSY-EC in TSB-YE medium were incubated for 24 h, with shaking at 200 rpm and 30 °C. Aliquots of the grown *P. polymyxa* cultures were centrifuged at 7500 rpm for 7 min, and the cell-free supernatants were harvested. The supernatants were filtered through a 0.45 µm membrane filter, and the resulting cell-free filtrates were collected. AI-2 signals, if produced by the *P. polymyxa* strains, were expected to be present in the cell-free filtrate. Aliquots of *V. harveyi* culture were dispensed in a 96-well black-bottom microtiter plate (Corning, Tewksbury, MA, USA). The *V. harveyi* culture was incubated for 24 h at 30 °C with 10% *P. polymyxa* strains cell-free filtrates. The optical density at 600 nm (OD_600_) and luminescence of *V. harveyi* were measured after 24 h of incubation using a microplate reader (SpectraMax 384 Plus; Molecular Devices, San Jose, CA, USA) and luminescence plate reader (Wallac Victor 3; PerkinElmer Life and Analytical Sciences, Shelton, CT, USA), respectively. Luminescence signals from *V. harveyi* were normalized to the bacterium’s cell density (OD_600_) and reported as relative luminescence units (RLUs)/OD_600_.

### 5.6. Impact of Autoinducer (AI-2) Quorum Sensing on Paenibacillin Production

To determine if AI-2 quorum sensing impacts paenibacillin production, D-ribose was used to inhibit AI-2 signaling activity in *P. polymyxa* strains, and the changes in paenibacillin production were monitored. D-Ribose is an AI-2 inhibitor, and it is structurally similar to AI-2’s furanosyl borate diether form. This structural similarity creates competition between D-ribose and AI-2, which allows D-ribose to inhibit AI-2 signals [41]. D-ribose was prepared at these concentrations: 0, 25, 50, 100, and 200 mM. These solutions were filter sterilized using 0.45 µm syringe filters (Millex HV Durapore PVDF; Merck Millipore, Ltd., Cork, IRL). An overnight culture of *P. polymyxa* OSY-DF or OSY-EC (1% seed culture) was used to inoculate fresh TSB-YE medium. D-ribose solutions were added to the inoculated media to achieve the concentrations mentioned earlier. Inoculated media were incubated at 30 °C for 24 h at a 200 rpm shaking condition. After 24 h of incubation, *P. polymyxa* cell-free filtrates were obtained by centrifugation at 7500 rpm for 7 min and filtration as mentioned previously. Cell-free filtrates were stored at −20 °C until further analysis. The antimicrobial activity of the cell-free filtrate containing paenibacillin was tested using the agar diffusion bioassay, which is described later. *V. harveyi* culture was incubated with 10% of *P. polymyxa* strains cell-free filtrates at 30 °C for 24 h. The luminescence and optical density (OD_600_) of these treated *V. harveyi* cultures were measured after 24 h of incubation using a luminescence plate reader and a microplate reader, respectively, as described earlier.

Additional experiments were performed to distinguish the effects of high carbohydrate concentrations from those associated with AI-2 inhibition. For these experiments, *P. polymyxa* OSY-DF and OSY-EC were grown in TSB-YE supplemented with 0, 100, or 200 mM D-ribose or 200 mM sucrose. Bacterial growth was monitored by measuring OD_600_ at 0, 2, 4, 8, 12, and 24 h. After 24 h, cell-free filtrates were prepared as described above and evaluated for antimicrobial activity and AI-2 signaling activity using *V. harveyi* as described above. To determine whether D-ribose or sucrose directly affected the *V. harveyi* BB170 reporter assay, *V. harveyi* BB170 was also treated with 10% (*v*/*v*) of TSB-YE containing 0, 25, 50, 100, or 200 mM D-ribose or 200 mM sucrose without *P. polymyxa* inoculation.

### 5.7. Bioassay for Measuring Paenibacillin Concentration

Detection and semi-quantification of the antimicrobial paenibacillin were achieved using the soft agar diffusion bioassay [42]. Previous characterization of the antimicrobial compounds produced by *P. polymyxa* OSY-DF using HPLC and LC-MS identified paenibacillin as the antimicrobial responsible for its anti-Gram-positive activity [7,23].

Therefore, anti-*Listeria* activity was used as an indicator of paenibacillin production in this study. Briefly, a 10 mL sterile molten tryptic soy soft agar (0.75% agar) was inoculated with a diluted (1:1000) overnight culture of *L. innocua,* an indicator strain, and mixed gently. The inoculated soft agar was poured onto a TSA agar in a Petri plate. Once the soft agar solidified, 5 µL of cell-free filtrate, collected at different STPs, were spotted onto the indicator lawn. The plates were incubated overnight at 37 °C and the presence of clear inhibitory areas were indicative of paenibacillin production. Additionally, the diameters (mm) of inhibition areas, corresponding to the cell-free filtrate spots, were measured from the digital Petri plate images using ImageJ version 1.54p software after image scale calibration. For the temporal experiment completed to evaluate the potential role of the Agr-like QS system in paenibacillin biosynthesis, paenibacillin concentration was estimated semi-quantitatively using a previously described dilution bioassay based on the minimum inhibitory concentration (MIC) of purified paenibacillin against *L. innocua* ATCC 33090 (4 µg/mL) [42]. Briefly, cell-free filtrate was serially two-fold diluted, and 10 µL of each dilution was spotted onto TSA plates overlaid with soft TSA seeded with *L. innocua*. Following incubation at 37 °C overnight, the last dilution showing visible inhibition was used to estimate paenibacillin concentration [42] as follows.

$$\mathrm{Paenibacillin}\;\mathrm{in}\;\mathrm{crude}\;\mathrm{extract}\;(\mu g/\mathrm{mL})\;=\;\frac{4\;\mu g}{\mathrm{mL}}\times \frac{1}{\mathrm{Dilution}\;\mathrm{factor}\;\mathrm{for}\;\mathrm{last}\;\mathrm{dilution}\;\mathrm{with}\;\mathrm{inhibition}}$$

### 5.8. Statistical Analysis

Each experiment involving gene transcription analysis of the paenibacillin BGC was completed with four technical replicates and was independently repeated three times (*n* = 3). The relative gene transcription results were represented as the mean fold change with error bars representing standard deviations calculated based on ΔΔCt values. Each experiment for the AI-2 study was repeated independently four times, and the results are expressed as the means ± standard deviations of the repeats. All results of experiments with continuous datasets, including the AI-2 detection and inhibition study, and the antimicrobial production analysis, were tested for normality using the Shapiro–Wilk test prior to performing comparative statistical tests. For the comparative statistical test, a parametric one-way analysis of variance (ANOVA), combined with a Tukey test, was performed using a commercial statistical software (GraphPad Prism 10.6.1). Pearson correlation analysis was performed to determine the temporal transcriptional association between Agr-like QS genes and paenibacillin biosynthetic *(pae*) genes. Mean relative transcriptional fold change, measured at 4, 8, 12, and 24 h of incubation, was used for each gene. Pearson correlation coefficients (*r*) were calculated for all Agr-Pae gene pairs. *p*-values were adjusted for multiple comparisons using the Benjamini–Hochberg false discovery rate procedure. The statistical analysis and heatmap visualization for the Pearson correlation analysis were performed in R version 4.1.3 using the “pheatmap” package.

## 6. Conclusions

Two quorum sensing systems in paenibacillin-producing *P. polymyxa* strains were explored as potential contributors to the biosynthesis of the antimicrobial peptide. The putative Agr-like quorum sensing genes and the biosynthetic genes are co-located within the paenibacillin BGC. The temporal transcriptional patterns of these two groups of genes suggest a potential association between the Agr-like quorum sensing system and paenibacillin biosynthesis. Although AI-2 activity was detected in both *P. polymyxa* strains, inhibition of AI-2 signaling did not result in a measurable reduction in paenibacillin antimicrobial activity under the conditions evaluated, suggesting that AI-2 quorum sensing may not contribute to paenibacillin biosynthesis under the conditions tested. These findings provide the basis for future functional studies to validate the role of quorum sensing in paenibacillin biosynthesis and facilitate strategies to enhance paenibacillin production.

## Figures and Tables

**Table 1:** Primer sequence for paenibacillin biosynthesis genes used for the real-time PCR in this study. The primers are based on alignment with the paenibacillin biosynthesis gene cluster (GenBank accession: JQ728481.1) from *Paenibacillus polymyxa* OSY-DF.

Paenibacillin Biosynthesis Genes	Gene Function	Primer Sequences (5′-3′)	PCR Products (bp)
*paeA*	Paenibacillin prepropeptide	F: GCTATGAGGCCAGTGAGCTT	111
		R: TGGAACAAGAACCGGTACAGA	
*paeP*	Peptidase	F: CGATTACGTCGGGCATGGTA	260
		R: CATTGGCGCAGTCAACCAAA	
*paeB*	Dehydratase	F: GCCGGTTCAATTCGTGCTTT	224
		R: TCGAGGGGCTGGAGACATAA	
*paeC*	Cyclase	F: ATGCAGGCTTGCTCCTGATT	190
		R: ACACCTGCACATCCATCGAG	
*paeI*	Putative immunity protein	F: ACAGCCGAATGTGTCGGAAT	204
		R: TTTCCGCCCTCTCTTCAACC	
*paeT*	Transporter	F: TTACACTGATTTCCGCCGCT	161
		R: GAGGAGCAGAGAAGACACGG	
*agrA*	Response regulator	F: CGACGCTCTGTCTGCTTTCT	151
		R: AGTGCGATGACCACGAAAAC	
*agrB*	Accessory gene regulator	F: CCATACCCAGAGACTCAAGGC	77
		R: ACCCGAAACAAATCCCTCCTC	
*agrC*	Histidine kinase	F: TGGACGAGGGGATCAGAACT	186
		R: CAACTCCACGCTTCTTTGCC	
*agrD*	Autoinducing peptide	F: CACGGTCATCTGTCTCCGAT	238
		R: TTTTGTGGGGAACACCCGTT	
*paeN*	Putative acetylase	F: TGTGGTGCCTTACGGATTGG	147
		R: ACGCAAAGAGCTGAAATCGC	
*16S rRNA*	Housekeeping gene	F: CTGCTTAGAGTGCCCAGCTT	189
		R: ACGCGAAGAACCTTACCAGG	

## Data Availability

The data are included within the manuscript and Supplementary Materials.

## Supplementary Materials

The following supporting information can be downloaded at https://www.mdpi.com/article/10.3390/antibiotics15100976/s1. Figure S1: Preliminary experiments showing *Paenibacillus polymyxa* OSY-DF growth (panel a) and paenibacillin antimicrobial activity (panel b) during 36 h of incubation in tryptic soy broth, supplemented with yeast extract, at 30 °C and shaking at 200 rpm. The growth was measured as changes in population counts (log_10_ CFU/mL) and the antimicrobial activity was presented as diameters of inhibition areas against *Listeria innocua* using an antimicrobial spot bioassay. Each data point is presented as the mean of 3 replicates, and each error bar represents ± SD; Figure S2: Preliminary experiments showing paenibacillin antimicrobial activity of cell-free filtrates collected during 36 h of incubating *Paenibacillus polymyxa* OSY-DF in growth medium. The antimicrobial activity was observed as areas of inhibition against *Listeria innocua* using an antimicrobial spot bioassay; Figure S3: Growth of *Paenibacillus polymyxa* OSY-DF (panel a) and OSY-EC (panel b) during 24 h of incubation in tryptic soy broth supplemented with yeast extract (TSBYE) and different concentrations of D-ribose (0, 100, and 200 mM) or sucrose (200 mM). Growth was measured as changes in optical density (OD_600_) at 0, 2, 4, 8, 12, and 24 h. Each data point is presented as the mean of 3 replicates, and each error bar represents ± SD; Figure S4: Determination of quorum sensing AI-2 activity in *Paenibacillus polymyxa* OSY-DF and OSY-EC, measured as relative luminescence units (RLUs)/OD600 reported by *Vibrio harveyi* BB170 treated with cell-free extracts (CEs) from strains grown in 0, 100, or 200 mM D-ribose or 200 mM sucrose for 24 h. Data are presented as the means of three replicates ± SD. Different letters indicate significant differences among treatments (*p* < 0.05); Figure S5: Antimicrobial activity, measured as diameters of inhibition areas against *Listeria innocua* using an agar diffusion bioassay, of cell-free extracts (CEs) from *Paenibacillus polymyxa* OSY-DF and OSY-EC grown in 0, 100, or 200 mM D-ribose or 200 mM sucrose for 24 h. Data are presented as the means of three replicates ± SD. Asterisks (*) indicate significant differences between OSY-DF and OSY-EC within each treatment (*p* < 0.05). Different letters (A, B, and C) indicate significant differences among treatments (*p* < 0.05); Figure S6: Effect of D-ribose and sucrose on the *Vibrio harveyi* BB170 AI-2 reporter assay, measured as relative luminescence units (RLUs)/OD_600_. *V. harveyi* BB170 was treated with 10% of tryptic soy broth with yeast extract (TSBYE) supplemented with 0, 25, 50, 100, or 200 mM D-ribose or 200 mM sucrose, without *Paenibacillus polymyxa*. Data are presented as the means of three replicates ± SD. “ns” indicates no significant differences among treatments (*p* > 0.05); Table S1: Presence of AI-2 system genes in *Paenibacillus* polymyxa strains (OSY-DF and OSY-EC) tested in this study. Genes were identified using the NCBI’s Basic Local Alignment Search Tool (BLAST version 2.17.0) against the OSY-DF genome (AIPP01000001) and OSY-EC (JABBXE010000001); Table S2: Putative restriction-modification (R-M) system genes identified in *Paenibacillus polymyxa* OSY-DF. Genes were identified from the annotated *P. polymyxa* OSY-DF genome using Bakta v1.11.3 (database v6.0).
