# Supplementary material for: Growth-Dependent Paenibacillin Biosynthesis and Its Association with an Agr-like Quorum Sensing System in Paenibacillus polymyxa
Source: Antibiotics (Basel). 2026 Oct 3;15(10):976. doi: 10.3390/antibiotics15100976 (PMC13700820; doi:10.3390/antibiotics15100976)
Supplement: Supplementary file 1 [file antibiotics-15-00976-s001.zip › antibiotics-4559508-supplementary.pdf]

Supplemental material

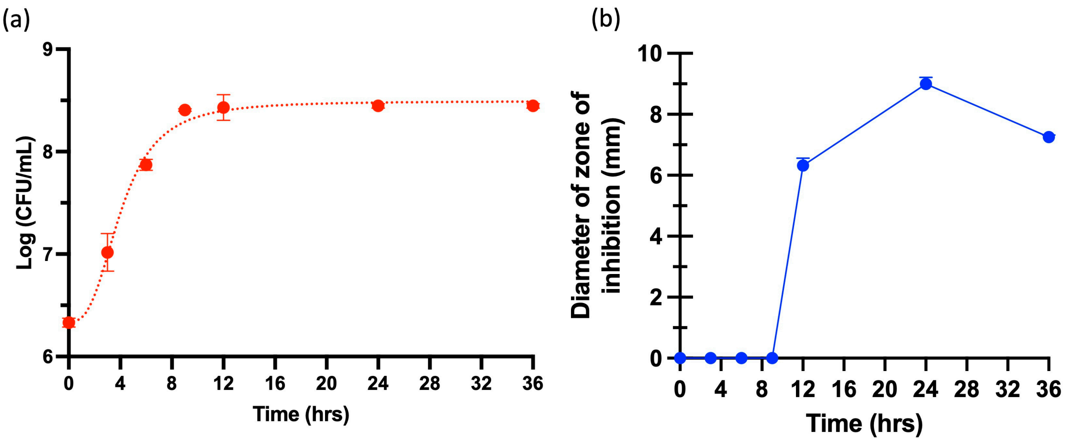

Figure S1: Preliminary experiments showing *Paenibacillus polymyxa* OSY-DF growth (panel a) and paenibacillin antimicrobial activity (panel b) during 36 h of incubation in tryptic soy broth, supplemented with yeast extract, at 30°C and shaking at 200 rpm. The growth was measured as changes in population counts ($\log_{10}$ CFU/mL) and the antimicrobial activity was presented as diameters of inhibition areas against *Listeria innocua* using antimicrobial spot bioassay. Each data point is presented as the mean of 3 replicates, and each error bar represents ± S.D.

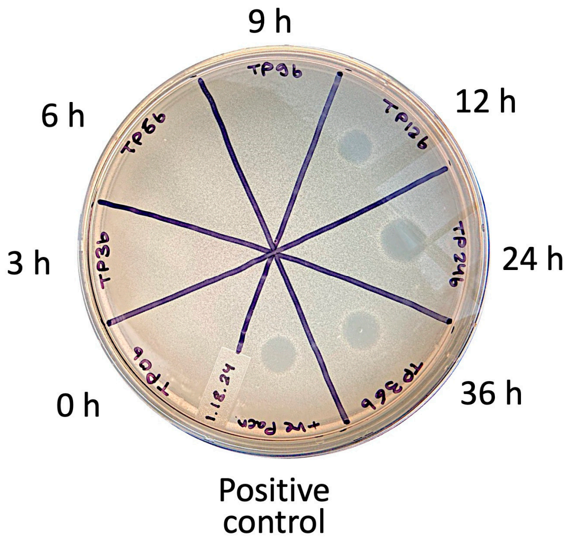

Figure S2: Preliminary experiments showing paenibacillin antimicrobial activity of cell free filtrates collected during 36 h of incubating *Paenibacillus polymyxa* OSY-DF in growth medium. The antimicrobial activity was observed as areas of inhibition against *Listeria innocua* using antimicrobial spot bioassay

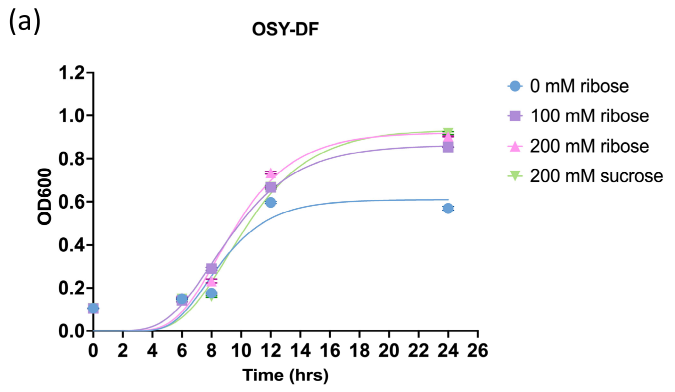

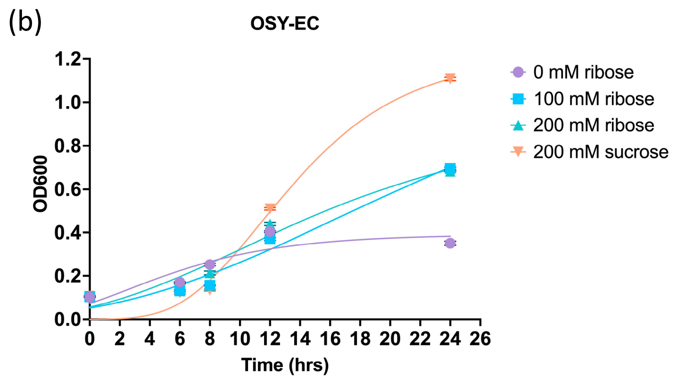

Figure S3: Growth of *Paenibacillus polymyxa* OSY-DF (panel a) and OSY-EC (panel b) during 24 h of incubation in tryptic soy broth supplemented with yeast extract (TSBYE) and different concentrations of D-ribose (0, 100, and 200 mM) or sucrose (200 mM). Growth was measured as changes in optical density (OD_{600}) at 0, 2, 4, 8, 12, and 24 h. Each data point is presented as the mean of 3 replicates, and each error bar represents ± S.D.

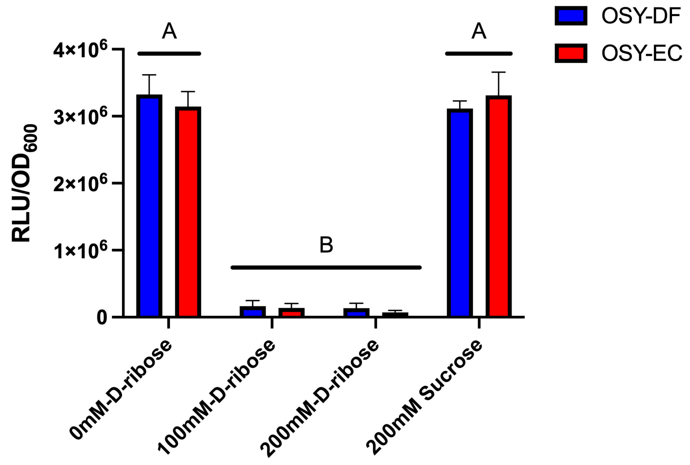

Figure S4: Determination of quorum sensing AI-2 activity in *Paenibacillus polymyxa* OSY-DF and OSY-EC, measured as relative luminescence units (RLU)/ OD_{600} reported by *Vibrio harveyi* BB170 treated with cell-free extracts (CE) from strains grown in 0,

100, or 200 mM D-ribose or 200 mM sucrose for 24 h. Data are presented as the means of three replicates ± SD. Different letters indicate significant differences among treatments ($p < 0.05$).

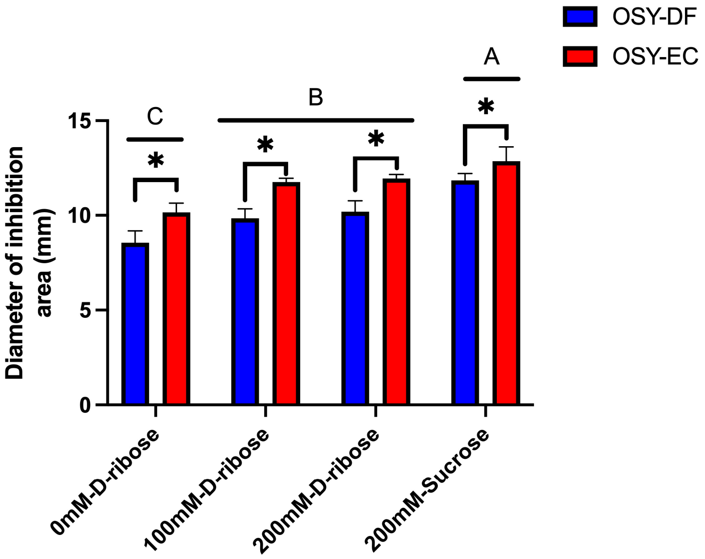

Figure S5: Antimicrobial activity, measured as diameters of inhibition areas against *Listeria innocua* using an agar diffusion bioassay, of cell-free extracts (CE) from *Paenibacillus polymyxa* OSY-DF and OSY-EC grown in 0, 100, or 200 mM D-ribose or 200 mM sucrose for 24 h. Data are presented as the means of three replicates ± SD. Asterisks (*) indicate significant differences between OSY-DF and OSY-EC within each treatment ($p < 0.05$). Different letters (A, B, and C) indicate significant differences among treatments ($p < 0.05$).

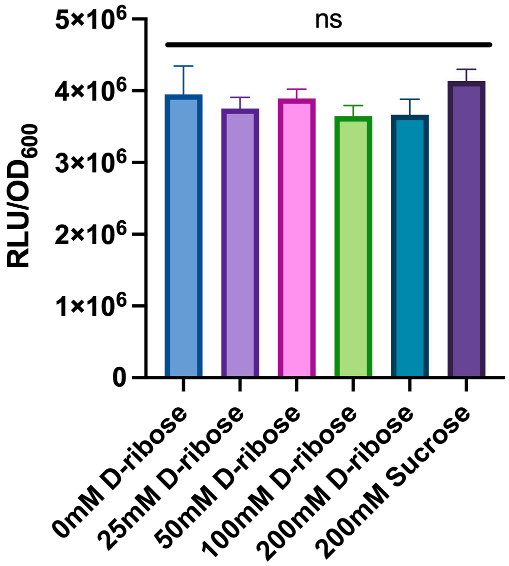

Figure S6: Effect of D-ribose and sucrose on the *Vibrio harveyi* BB170 AI-2 reporter assay, measured as relative luminescence units (RLU)/OD600. *V. harveyi* BB170 was treated with 10% of tryptic soy broth with yeast extract (TSBYE) supplemented with 0, 25, 50, 100, or 200 mM D-ribose or 200 mM sucrose, without *Paenibacillus polymyxa*. Data are presented as the means of three replicates ± SD. "ns" indicates no significant differences among treatments ($p > 0.05$).

Table S1. Presence of AI-2 system genes in *Paenibacillus polymyxa* strains (OSY-DF and OSY-EC) tested in this study. Genes were identified using the NCBI's Basic Local Alignment Search Tool (BLAST) against the OSY-DF genome (AIPP01000001) and OSY-EC (JABBXE010000001).

**Genes**	**OSY-DF**	**OSY-EC**
luxS	Present	Present
luxR	Present	Present
rbsB	Present	Present

Table S2. Putative restriction-modification (R-M) system genes identified in *Paenibacillus polymyxa* OSY-DF. Genes were identified from the annotated *P. polymyxa* OSY-DF genome using Bakta v1.11.3 (database v6.0).

**Locus tag**	**Genomic coordinates (bp)**	**Strand**	**Predicted protein**	**RefSeq accession**	**Putative function**
**CGKLGC_01291**	180,975–181,469	−	MvaI/BcnI family restriction endonuclease	WP_016819823.1	Restriction endonuclease
**CGKLGC_01293**	181,906–183,267	+	Cytosine-specific methyltransferase	WP_016819821.1	DNA methylation/ modification
**CGKLGC_01384**	273,076–275,007	−	Type III restriction endonuclease subunit M	WP_016819302.1	R-M-associated modification component
**CGKLGC_02268**	1,237,572–1,238,315	+	Restriction endonuclease	WP_016822541.1	Restriction endonuclease
**CGKLGC_03297**	2,379,863–2,380,195	+	Restriction endonuclease subunit S	WP_013372833.1	Sequence-specificity component
